# The clinical significance of atypical indirect immunofluorescence patterns on primate cerebellum in paraneoplastic antibody screening

**DOI:** 10.1186/s13317-019-0116-6

**Published:** 2019-07-25

**Authors:** Joris Godelaine, Xavier Bossuyt, Koen Poesen

**Affiliations:** 1 Department of Neurosciences, Laboratory for Molecular Neurobiomarker Research, KU Leuven (University of Leuven), Herestraat 49, 3000 Leuven, Belgium; 2 Laboratory Medicine, University Hospitals Leuven, UZ Herestraat 49, Leuven, Belgium; 3 Department of Microbiology and Immunology, Clinical and Diagnostic Immunology, KU Leuven (University of Leuven) Leuven, Herestraat 49, Leuven, Belgium

**Keywords:** Paraneoplastic neurological syndromes, Antineuronal antibodies, Indirect immunofluorescence, Primate cerebellum, Screening assay

## Abstract

**Purpose:**

Screening for paraneoplastic antibodies is often performed by means of indirect immunofluorescence on primate cerebellar slices. However, *atypical* immunofluorescence patterns, i.e. patterns that are not specifically related to paraneoplastic antibodies, are often reported. The clinical significance of these patterns is not clear. Therefore, the purpose of this study was to determine the significance and diagnostic value—in terms of a paraneoplastic neurological syndrome or other neurological disease being diagnosed in the patient—of such atypical immunofluorescence screening patterns on primate cerebellum.

**Methods:**

This study is a retrospective single center study including atypical indirect immunofluorescence screening patterns of patients with a negative or absent typing assay for intraneuronal and anti-amphiphysin paraneoplastic antibodies. Patients with a positive typing assay or without final diagnosis were excluded. Included patients were grouped according to (i) reported immunofluorescence pattern and (ii) established diagnosis, after which contingency table analyses were performed to investigate an interrelation between reported pattern and diagnostic group.

**Results:**

In 3.7% of cases, patients with an atypical pattern obtained a final diagnosis of a paraneoplastic neurological syndrome. The presence of atypical patterns was more prominent in patients with epilepsy or peripheral neuropathies (*p*_*Monte Carlo simulation*_= 0.026), without, however, adding any diagnostic information.

**Conclusions:**

An atypical indirect immunofluorescence pattern on primate cerebellum in the screening for paraneoplastic antibodies has only very minor relevance with respect to paraneoplastic neurological syndromes or any other neurological disease, recommending clinicians to interpret the results of positive screening assays for such antibodies with care.

**Electronic supplementary material:**

The online version of this article (10.1186/s13317-019-0116-6) contains supplementary material, which is available to authorized users.

## Introduction

Paraneoplastic neurological syndromes (PNS) are remote effects of cancer not caused by tumor growth, infiltration, metastasis or chemotherapy [1, 2]. They are predominantly the result of an autoimmune process, mediated by T-lymphocytes and/or antineuronal antibodies (AN Ab) [1, 3]. The current hypothesis is that the immune response is directed against neuronal antigens ectopically expressed by the tumor [1, 2, 4]. Often, PNS and corresponding Ab surface before a tumor is discovered [5–9]. Therefore, it is important to meticulously search for AN Ab since they might urge clinicians to initiate oncological screening [10, 11]. Moreover, since some Ab are closely linked with a certain PNS, detection of AN Ab facilitates the diagnosis of that specific PNS.

**Fig. 1:**
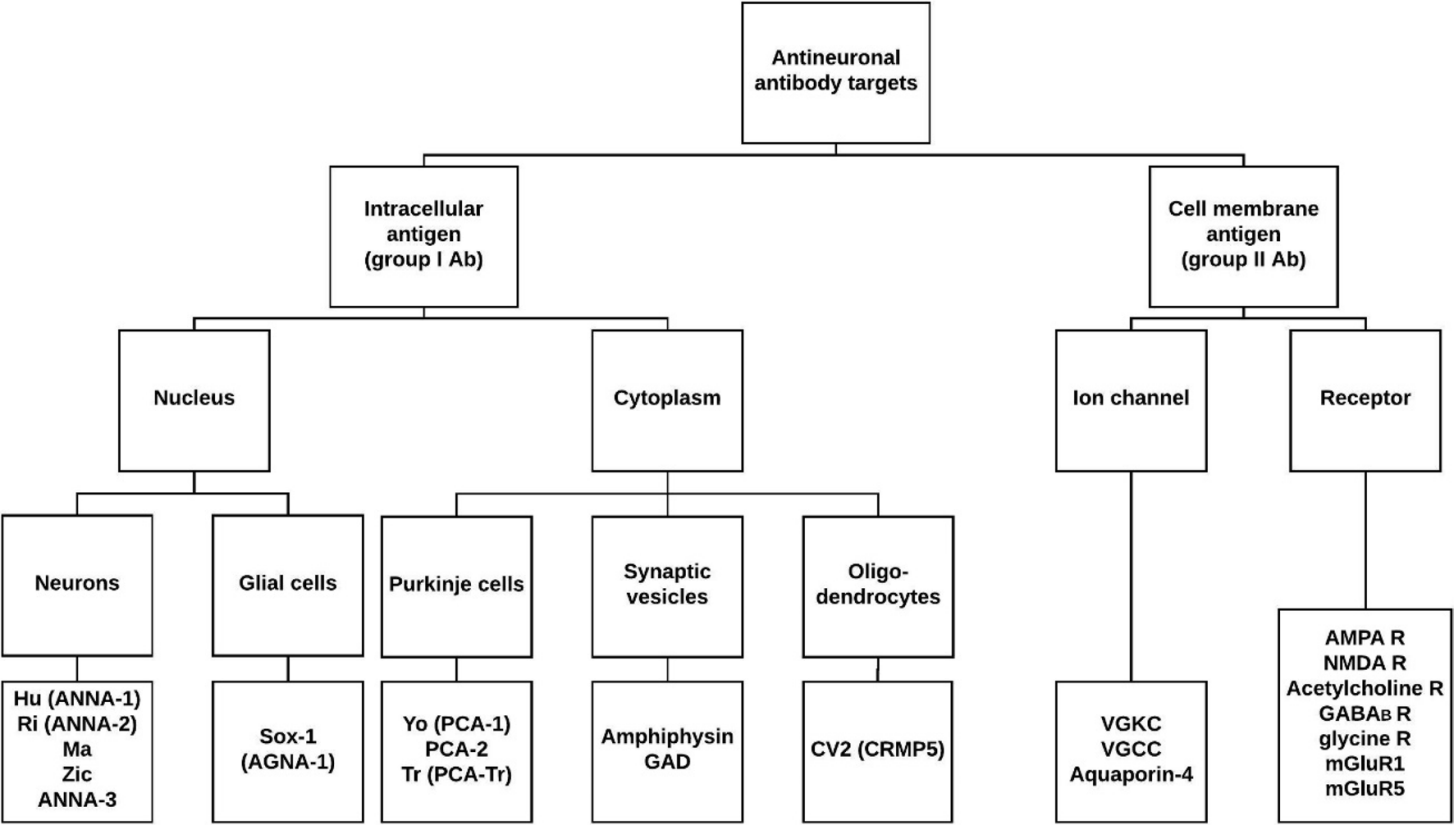
Location of antineuronal antibody targets. Antibodies directed against intracellular antigens (excluding anti-GAD antibodies) generate specific indirect immunofluorescence patterns on primate cerebellum. R, receptor. Illustration made with Lucidchart (http://www.lucidchart.com)

Based upon targeted antigen location, AN Ab can be divided into two subgroups: intracellular (group I) or on the cell membrane (group II) (Fig. 1) [1, 12, 13]. Their detection is classically performed by screening assays such as indirect immunofluorescence (IIF) screening on primate cerebellum, followed by typing assays such as line blots or cell-based assays [1, 13–16]. Intraneuronal and anti-Amphiphysin Ab (Fig. 1), the most often paraneoplastic (PN) Ab, show *typical* IIF patterns on primate cerebellum such as positive neuronal nuclei (anti-Hu) or coarse granular staining of cytoplasm of Purkinje cells (anti-Yo) [1, 12, 13, 17]. However, atypical patterns that are not specifically related to a PN Ab (i.e. negative on typing for intraneuronal and anti-Amphiphysin Ab), are also reported. For example, the Purkinje cell layer can show positivity without a specific pattern (coarse granular staining of Purkinje cell cytoplasm) being reported, while in e.g. the molecular layer a ‘pan-layer’ fluorescence instead of a specific pattern (e.g. dot-like fluorescence or positivity of neuronal nuclei) is often seen. The clinical significance of such patterns is not yet known. An overview of antineuronal antibodies with their associated fluorescence patterns, neoplasms and clinical features can be seen in Table 1.

**Table 1:** Fluorescence patterns, neoplasms and clinical features/syndromes of antineuronal antibodies

Antibody	Fluorescence pattern	Associated neoplasm	Associated clinical feature/syndrome
*Anti-Hu*^a,b^	Neuronal nuclei (CNS and PNS), granular pattern	SCLC, thymoma, neuroblastoma	PEM, limbic/cortical/brainstem encephalitis, PCD, PSN, myelitis, autonomic dysfunction
*Anti-Ri*^b^	Neuronal nuclei (CNS), granular pattern	Lung, breast	Brainstem encephalitis, PCD, opsoclonus-myoclonus
*Anti-Ma*	Nerve cell nucleoli	Lung, breast, germ cell tumor of testis	LE, hypnogogic hallucination, cerebellar/brainstem syndrome
*Anti-Zic4*	Nuclei of granular layer, ANA-like pattern	SCLC	LE, cerebellar/brainstem syndrome
*ANNA-3*	Nuclei of PC	Lung, upper airway	Cerebellar ataxia, myelopathy, sensory/sensorimotor neuropathy, myelopathy
*Anti-Sox-1*	Bergmann glia nuclei in the PCL	SCLC	LEMS, PCD, LE, sensory/sensorimotor neuropathy
*Anti-Yo*	PC cytoplasm, coarse granular pattern	Ovaria, breast, endometrium	PCD
*Anti-Tr*	PC cytoplasm, coarse granular pattern + ML, dot-like pattern	Hodgkin’s lymphoma	PCD, limbic encephalopathy
*PCA-2*	PC cytoplasm, extending into dendrites	SCLC	Brainstem/limbic encephalitis, PCD, LEMS, motor neuropathy
*Anti-Amphiphysin*	Presynaptic nerve terminals, intensity ML > GL	Lung, breast	SPS, PEM
*Anti-CV2*	ML (mostly), sand-like pattern	SCLC, thyroid gland, kidney, thymoma	PEM, PCD, chorea, optic/peripheral neuropathy, myelopathy
Anti-GAD	Presynaptic nerve terminals	Thymoma, others	SPS, MFS, LE, cerebellar ataxia, epilepsy
Anti-VGCC	ML	SCLC, lung, breast, ovarian	LEMS, cerebellar degeneration
Anti-VGKC	ML + GL	SCLC, thymoma	LE, PCD, parkinsonism, tremor, chorea, sensorimotor neuropathy, dyssomnia, hyperphagia, gastrointestinal dysmotility
Anti-aquaporin 4	Multiple layers (perivascular pattern)	Thyroid gland, thymoma	Neuromyelitis optica
Anti-NMDA R	GL	Ovarian teratoma	Psychiatric features, memory loss, orofacial dyskinesia, catatonic state, central hypoventilation, abnormal posturing
Anti-AMPA R	ML + GL	SCLC, thymoma, breast	LE, atypical psychosis
Anti-GABA_B_ R	ML + GL	SCLC	LE
Anti-glycine R	Neuropil staining	Lung	PERM
Anti-mGluR1	PC cytoplasm	Hodgkin’s lymphoma	PCD

*ANA* antinuclear antibodies, *CNS* central nervous system, *GL* granular layer, *LE* limbic encephalitis, *LEMS* Lambert-Eaton myasthenic syndrome, *MFS* Miller–Fisher syndrome, *ML* molecular layer, *PC* Purkinje cell, *PCD* paraneoplastic cerebellar degeneration, *PCL* purkinje cell layer, *PEM* paraneoplastic encephalomyelitis, *PERM* paraneoplastic encephalomyelitis with rigidity and myoclonus, *PNS* peripheral nervous system, *PSN* paraneoplastic sensory neuropathy, *R* receptor, *SCLC* small-cell lung cancer, *SPS* Stiff–Person syndrome

^a^Antibodies in italics generate specific patterns on IIF (i.e. patterns that allow to differentiate between antibodies present)

^b^Anti-Hu and Anti-Ri generate similar patterns (fluorescence of neuronal nuclei) but anti-Ri only stains nuclei of the central nervous system

According to our experience, an atypical IIF pattern is reported in about 90% of positive screening assays, not followed by a positive typing assay (line blot). Therefore, this retrospective study aimed to determine, in regard to PNS, the clinical significance of an *atypical* IIF pattern in the absence of a positive line blot for specific pattern-generating group I Ab. Furthermore, we evaluated, if any, the association between atypical IIF patterns on cerebellar slices on the one hand and particular diseases on the other hand.

## Methods

### Compliance with ethical standards

This retrospective study was approved by the local ethical committee of the University Hospitals of Leuven (file number S59935).

### Sample analysis

Serum and/or cerebrospinal fluid (CSF) samples of patients suspected of having a PNS are routinely outsourced for screening and typing to an experienced partner laboratory in Luxembourg (Laboratoires Ketterthill—LLIP, Belvaux, Luxembourg). IIF screening was performed upon diluted serum samples (1:10) and undiluted CSF samples with the Nova Lite^®^ Cerebellum kit (Inova diagnostics Inc., San Diego, USA), on cryostat frozen sections of primate cerebellum. Upon screening assay positivity, sample typing was performed by means of the EUROLINE Neuronal Antigen Profile 12^®^ line blot assay (Euroimmun AG, Luebeck, Germany), which tests for Ab directed against the following antigens: Amphiphysin, CV2, Ma/Ta, Ri, Yo, Hu, recoverin, Sox1, titin, Zic4, GAD and Tr. Results were sent by post and manually introduced into the laboratory informatics system.

### Inclusion criteria and data retrieval

To determine the clinical significance of an atypical IIF pattern in regard to PNS, patients included in this retrospective study had to meet three criteria: an atypical IIF screening pattern on primate cerebellar slices (i.e. not specifically related to a PN Ab, Table 1), a negative or absent line blot typing result for specific-pattern generating Ab (intraneuronal and anti-Amphiphysin Ab, Fig. 1) and having received a final diagnosis. Screening and typing results from the period of January 2009 to May 2017 were retrospectively retrieved from the laboratory informatics system in May 2017. 2009 was the year the currently used screening assay was implemented, May 2017 the end date of the study. Further relevant clinical information was retrieved from the laboratory informatics system as well, such as information regarding the IgG index, the presence of oligoclonal bands and other AN Ab (anti-GAD and group II Ab) assay results.

Final diagnoses of patients were established by neurologists from the University Hospital of Leuven during routine clinical practice based upon clinical symptoms, laboratory results and imaging data. Diagnoses were retrospectively retrieved for this study by the authors through investigation of individual patients’ medical reports. When medical reports were not conclusive about the certainty of the diagnosis, the treating clinician was consulted. Patients without a certain final diagnosis were excluded.

### Patient classification

To enable hypothesis testing of a possible correlation between reported pattern and diagnosis, each patient was assigned to two groups. First, each patient was assigned to one of the ‘pattern groups’, based upon which cerebellar layer or pattern was reported to be positive for the patient in question. Patterns reported positive in five or less patients—including ‘blood vessels’, ‘grey matter’, ‘white matter’, ‘neuropil’, ‘nucleus of neurons’ and ‘synapses of neurons’—were grouped into the ‘minor patterns’ group for statistical reasons. Patients with multiple layers or patterns reported positive were grouped into the ‘multiple patterns reported’ group. When the screening assay was reported positive but no pattern was specified, patients were assigned to the ‘no pattern reported’ group and excluded for hypothesis testing.

The second group patients were assigned to, was according to the patient’s final diagnosis: based on the International Classification of Diseases 11th Revision for Mortality and Morbidity Statistics (ICD-11 MMS) published by the World Health Organization, diagnoses were grouped into ‘diagnostic groups’. Medical files of patients considered to have a PNS by the consulting neurologist, were examined by the authors to ascertain compliance with the diagnostic criteria for PNS and subsequently assigned to the PNS group [7, 10, 11, 18, 19]. Diseases without a clear alignment to any subdivision of the ICD-11 MMS were assigned to their respective diagnostic groups based upon authors’ consensus after consulting clinical and laboratory results.

### Reported IIF pattern—diagnosis correlation

A non-parametric R × C contingency table containing pattern groups (rows) versus diagnostic groups (columns) was constructed to test the hypothesis of a possible interrelation between reported atypical IIF pattern and patients’ final diagnosis. Assumptions for the Chi square (χ^2^) test for independence—an expected cell value of five or more in at least 80% of cells and an expected value of at least one in every cell [20]—were not met, while a ‘standard’ Fisher’s exact test could not be executed due to insufficient computing power for such an extensive table. Therefore, a Monte Carlo simulation (number of simulations = 200.000, 99% CI) of the Fisher’s Exact test was chosen to estimate the *p* value in the first contingency table analysis. When an interrelation was present, ‘standard’ Fisher’s Exact tests were executed for each pattern individually (i.e. pattern present or absent) versus all diagnostic groups (n = C) to determine whether the number of reported patterns in the combined diagnostic groups was statistically significant (e.g. 2 × C cross table analysis). When this second set of statistical analyses produced a significant result for a certain pattern, Fisher’s Exact tests were executed to determine whether the number of times that this particular pattern was reported in a certain individual diagnostic group was significantly different compared to other diagnostic groups (e.g. 2 × 2 cross table analysis). Bonferroni correction for multiple testing was applied for all ‘standard’ Fisher’s Exact tests. SPSS Statistical software (Version 25.0, IBM Corp., Armonk, US) was used for all analyses.

## Results

### Primary outcome analysis: correlation between atypical IIF patterns and final diagnosis

From January 2009 to April 2017, the IIF screening assay was performed 2415 times for 2126 patients, of which 261 patients tested positive. Of these 261 patients, 78 were excluded for not yet having received a final diagnosis and 21 for testing positive for specific pattern-generating group I Ab (intraneuronal and anti-Amphiphysin Ab) on the line blot typing assay (21 patients: six with anti-Yo, four with anti-Hu, three with anti-Ri, three with anti-CV2, two with anti-Amphiphysin, two with anti-SOX1 and one with both anti-Hu and anti-GAD65 Ab; all in blood except for the patient with both anti-Hu and anti-GAD65 Ab who tested positive on CSF). As such, 162 patients [90 females (55.6%), 72 males (44.4%); median age: 59.5 years; range 3–88 years] with a positive IIF screening (on serum, N = 154; on CSF, N = 3; on both, N = 5), negative or absent typing and a final diagnosis were included. A flowchart illustrating the patient inclusion/exclusion process can be seen in supplement (Additional file 1: Figure S1). Based on the patterns reported in the 162 included patients, seven pattern groups were established: granular layer positive, molecular layer positive, Purkinje cell layer positive, neurofilaments positive, minor patterns positive, multiple patterns reported and no pattern reported. For 16 patients, no IIF pattern was reported (i.e. reported solely as ‘positive’) and hence these patients were excluded for subsequent hypothesis testing.

Seventy-two different diagnoses were reported for the 162 included patients, and, in total, nine diagnostic groups were established (Table 2). The most prevalent diagnostic groups were the ‘epilepsy’ (n = 46; 28.4%) and the ‘peripheral neuropathies (n = 33; 20.4%) groups. Six out of 162 cases (3.7%) with an atypical IIF pattern obtained the diagnosis of a PNS, with two cases related to the presence of anti-GAD or group II AN Ab (Additional file 2: Table S1). These six patients retrospectively fulfilled the Graus et al. 2004 consensus criteria of ‘definite’ PNS [7]. Patients diagnosed by the consulting neurologist with a form of autoimmune encephalitis all complied with the Graus et al. 2016 autoimmune encephalitis consensus criteria [19]. Cancer was diagnosed in fifteen patients without, however, the presence of a ‘classical’ or ‘non-classical’ PNS as other factors were established as cause of the patient’s symptoms (e.g. metastatic seeding or tumor growth). Hence, since these patients presented with an oncological process but did not fulfill the consensus criteria for a PNS, they were assigned to the ‘tumor-related diagnosis’ group (Table 2) [7]. The contingency table established utilizing the pattern - and diagnostic groups can be seen in Table 3.

**Table 2:** Individual diagnoses of patients within each diagnostic group

(Auto-) immune-mediated diseases (18; 11.1%)	Epilepsy (46; 28.4%)	Inflammatory/infectious CNS diseases (13; 8%)	Movement disorders (14; 8.6%)	Neurocognitive disorders (14; 8.6%)	Neuromuscular diseases (3; 1.9%)	Peripheral (poly-) neuropathies (33; 20.4%)	PNS (6; 3.7%)	Tumor-related diagnoses (15; 9.3%)
ALPS^a^	Absence seizures	Encephalopathy	Cerebellar ataxia (4)	Alzheimer’s (4)	ALS (3)	CIDP (9)	Burkitt lymphoma: encephalitis	Adenocarcinoma: CIDP
Anti-VGKC LE (4)	Unspecified EP (8)	Irradiation leuko-encephalopathy	Cerebellar syndrome	Frontal network syndrome		Demyelinating motoric PNP	Hodgkin’s lymphoma: CD	Adenocarcinoma: MC
AI encephalitis (2)	Focal EP (5)	Meningitis	Cerebellar-pyramidal syndrome	Frontotemporal degeneration		Diabetic PNP (2)	Lung adenoma: anti-VGKC LE	Acute myeloid leukemia
AI PNP (2)	Generalized EP (8)	Myelitis transversa	Chorea-atotic syndrome	Frontotemporal dementia		Guillain-Barré syndrome (4)	Mammary carcinoma: cerebellar ataxia	Angiomyolipoma: de novo EP
Hypophysitis (lymphocytic)	Lesional EP (3)	Myelitis transversa + meningo-encephalitis	Multiple system atrophy (3)	Lewy body disease		Miller-Fisher syndrome	Small-cell lung cancer: LE	Cervix carcinoma: de novo EP
Multiple sclerosis	Temporal lobe EP (21)	Myelopathy (Vit. B12 deficiency)	PSP (2)	Multidomain mild cognitive impairment		Mononeuritis multiplex	Thymoma: anti-GAD LE	Ependymoma: compartment syndrome
Myasthenia gravis		N. Trigeminus failure	SPS (2)	Normal-pressure hydrocephalus		Motor PNP		Glioma
Myelitis transversa + NMO		Neurosarcoidosis		Psychosis (4)		Sensory PNP (2)		Lung carcinoma: MC (2)
PERM		Viral encephalitis (5)				Sensorimotor PNP (10)		Mammary carcinoma: hypermetropia and presbyopia
Polymyositis						SFNP (2)		Melanoma: myelitis
Ramsay Hunt syndrome								Neuroblastoma
Seronegative LE^b^ (2)								Ovarium carcinoma (2)
								Pancreas carcinoma: CIDP

*X:Y* diagnosis Y associated with cancer X, *AI* autoimmune, *ALPS* autoimmune lymphoproliferative syndrome, *ALS* amyotrophic lateral sclerosis, *CD* cerebellar degeneration, *CIDP* chronic inflammatory demyelinating polyneuropathy, *EP* epilepsy, *LE* limbic encephalitis, *MC* meningitis carcinomatosa, *NMO* neuromyelitis optica, *PERM* progressive encephalomyelitis with rigidity and myoclonus, *PNP* polyneuropathy, *PSP* progressive supranuclear palsy, *SFNP* small-fiber polyneuropathy, *SPS* Stiff–Person syndrome

^a^Number of patients diagnosed with the disease, 1 patient when not specified

^b^Diagnosis based on clinical symptoms and treatment effect

**Table 3:** Reported patterns versus diagnostic groups contingency table

	(Auto-) immune-mediated diseases	Epilepsy or seizures	Inflammatory/infectious CNS diseases	Movement disorders	Neurocognitive disorders	Neuromuscular diseases	Peripheral (poly)neuropathies	PNS	Tumor-related diagnoses	Total
Granular layer	6	14	2	0	5	1	17	1	8	54
Minor patterns	1	6	1	2	1	0	4	1	4	20
Molecular layer	3	3	2	4	4	2	2	2	1	23
Multiple patterns reported	2	10	1	3	2	0	5	2	2	27
Neurofilaments	0	1	2	2	0	0	1	0	0	6
Purkinje cell layer	0	7	5	0	1	0	3	0	0	16
Total	12	41	13	11	13	3	32	6	15	146

**Fig. 2:**
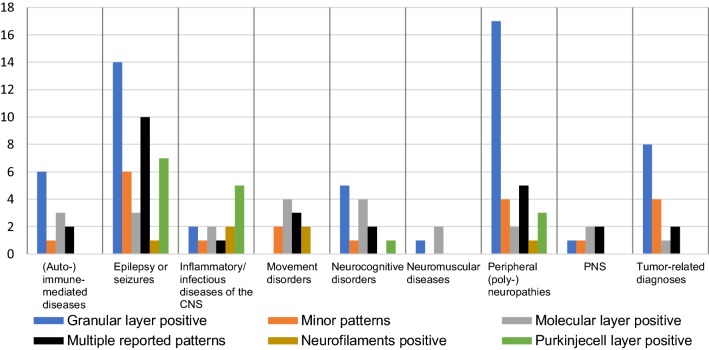
Number of reported immunofluorescence patterns for each diagnostic group. Illustration made with Microsoft Excel 2016

As depicted in Fig. 2, a large variety of different patterns were reported within the diagnostic groups. Interestingly, the atypical pattern ‘positive granular layer’ seemed to occur mostly in the epilepsy and peripheral neuropathy groups and, within those groups, this pattern was also the most prevalent one (Table 2 and Fig. 2). The Monte Carlo simulation of the Fisher’s Exact test on a group level demonstrated a significant different distribution of atypical IIF patterns among the different diagnostic groups [*p* = 0.026 (99% CI 0.025–0.027)]. However, the distribution of positivity or negativity for one particular layer among different diagnostic groups was not statistically different (all *p* > 0.0083 (Table 4), the Bonferroni corrected level of significance). As such, no further statistical analyses were performed to determine whether the number of times that a certain pattern was reported in a given diagnostic group differed significantly from other diagnostic groups. An elevated IgG index or the presence of oligoclonal bands was reported in respectively 27/162 and 14/162 patients (individual results not shown). However, a significant different distribution of these parameters among the pattern groups was not present, with *p*-values of respectively 0.733 and 0.091.

**Table 4:** *p*-values of the distribution of the result for a given pattern group among diagnostic groups

Pattern group	Fisher’s exact p-value^a^
Granular layer positive	0.015
Minor patterns positive	0.910
Molecular layer positive	0.019
Multiple reported patterns	0.856
Neurofilaments positive	0.210
Purkinje cell layer positive	0.090

Fisher’s exact tests were executed on a group level to determine if a certain layer was reported significantly more often to be positive

^a^Bonferroni correction for multiple testing:  Significant if p < 0.0083

### Secondary outcome analysis: presence of other AN Ab including surface Ab

To further comprehend the large number of patients with positive screening assays not receiving a PNS diagnosis (156 patients), the presence of other AN Ab (anti-GAD or group II Ab) as a cause of positivity was retrieved from the laboratory informatics system. 34 patients’ samples were not tested for the presence of any group II or anti-GAD Ab, while for the remaining patients at least one anti-GAD or group II Ab assay had been performed: 22 patients tested positive for anti-GAD (on line blot) or group II Ab (on cell-based assays), of whom two patients were diagnosed with a PNS (one anti-VGKC LE associated with a thymoma and one anti-GAD LE associated with lung adenoma; Table 2 and Additional file 2: Table S1). As such, 20 ‘non-PNS’ patients were positive for one of the following AN Ab: aquaporine-4 Ab (one patient), anti-GAD & anti-GABA_B_ receptor Ab (one patient), anti-GAD Ab (11 patients) and high titers of anti-VGKC Ab (nine patients; these Ab are now subdivided in anti-LGI1 and anti-CASPR2 Ab, but not at the time of clinical reporting of results). No group I or group II AN Ab were detected in the remaining 136 non-PNS patients, with the limitation that a broad screening for AN Ab was lacking in some patients (e.g. 34/162 were not tested for the presence of any group II AN Ab, anti-GAD presence was tested in 98 patients, anti-VGKC in 87 patients, anti-NMDA receptor in 76 patients).

## Discussion

In this study, we demonstrated that atypical IIF patterns on primate cerebellar slices are most often observed in neurological diseases other than PNS, including epilepsy and peripheral neuropathies. In only approximately 3.7% of cases, patients with an atypical IIF pattern were diagnosed with a PNS. Among the different disease groups, a different distribution of atypical patterns was seen (i.e. on a group level). This, however, did not result in any added diagnostic information as among different disease groups, a significant different distribution between positive and negative was absent for each and every pattern group. Indeed, within the same diagnostic group, multiple different layers were reported to be positive (Fig. 2).

Another possible cause for the clinically irrelevant results is the binding of atypical pattern generating AN Ab (i.e. anti-GAD or group II Ab) or non-AN Ab to antigens in primate cerebellum. This would lead to the positivity of certain layers, despite the absence of typical pattern-generating group I Ab (intraneuronal and anti-Amphiphysin Ab). Such an explanation has previously been suggested by Haukanes et al. [21]. In their study, some ADHD patients’ samples demonstrated a positive staining of the cytoplasm of Purkinje cells on IIF screening [21]. However, since typing assays were negative for most patients, the authors decided that the reported patterns were probably due to a non-specific binding of Ab to unknown antigens [21]. Of interest, our study observed the presence of AN Ab other than typical pattern-generating antibodies (i.e. anti-GAD and group II Ab) in 20 of the 156 ‘non-PNS’ patients. These Ab included anti-GAD, anti-GABA_B_, anti-VGKC and anti-aquaporine-4 Ab and most were searched for in the majority of patients (e.g. 98/162 for anti-GAD, 87/162 for anti-VGKC and 76/162 for anti-NMDA receptor Ab). As the cumulative prevalence of those antibodies—as reported by the large scale study of Dahm et al.—in a ‘neurological disease population’ (0.5% for anti-GAD and ≤ 0.1% for the other Ab) is clearly lower than seen in our cohort, the percentage of patients with anti-GAD or group II Ab might be enriched in a cohort of patients having an atypical IIF pattern on primate cerebellar slices [22]. Therefore, it might be recommended that if the clinical presentation correlates with the presence of an AN Ab but an atypical IIF pattern and a negative line blot are produced by the laboratory, the search for Ab might be extended to cover the cell-surface (group II) Ab as well. In such case, before considering assays such as cell-based assays to identify group II Ab, the IIF screening can be extended to hippocampal slices as group II Ab have been shown to generate more typical patterns on hippocampal slices [14]. As such, the pattern generated on the hippocampal slices can provide valuable information regarding the identity of the group II Ab present, and hence suggest which cell-based assay should be performed. Moreover, the combination of both hippocampus slices and cell-based assays can provide higher sensitivities and specificities than cell-based assays alone [23].

Some limitations were encountered in this study. Firstly, our study did not include paired serum and CSF samples for most patients, as for only five patients screening was performed on both serum and CSF (only on serum, N = 154; only on CSF, N = 3). Nevertheless, it would be of interest to learn whether an atypical IIF pattern seen in serum could be confirmed in CSF. If so, it could also be worthwhile to investigate whether such paired results are related to the presence of AN Ab or to the clinical diagnosis of PNS. A second limitation was encountered when investigating possible explanations for the positivity of IIF screening assays, in light of the absence of group I Ab (excluding anti-GAD). Our secondary outcome analysis provided evidence that the presence of AN Ab other than the typical-pattern generating Ab could be put forward as a possible explanation. However, since this was a retrospective study using results gathered in daily routine clinical practice, not all samples received the same extent of AN Ab screening (i.e. not all samples were tested on the presence of every known AN Ab). A probable explanation for this could be that the consulting neurologist did not consider group II Ab as a possible cause for symptoms seen in a patient and, hence, the neurologist did not request any analysis for group II Ab (including surface Ab screening. Nevertheless, an extensive AN Ab screening for each patient with a positive yet atypical IIF pattern would further contribute to the understanding of the significance of such atypical IIF patterns. Therefore, a follow-up study where for each sample an extensive search for all AN Ab is performed (including for group II Ab) in order to generate a more complete overview of patients’ positive layers and AN Ab present, would be interesting.

## Conclusion

A limited number of studies investigated the performance characteristics of the IIF screening on primate cerebellum. This was done in the context of the sensitivity and specificity of IIF on cerebellar slices for diagnosing PNS [4, 24, 25]. Our study, however, investigated the clinical significance of *atypical* IIF patterns on cerebellar slices, i.e. patterns that are not related to the presence of group I Ab (excluding anti-GAD Ab) and that are not followed by a positive line blot typing assay. Now, we show that such atypical IIF patterns are of limited clinical significance. Nevertheless, the enrichment of AN Ab (including anti-GAD, anti-VGKC and anti-GABA_B_ Ab) in a cohort of patients with atypical IIF patterns, calls for further investigation with respect to action required upon the presence of an atypical pattern on primate cerebellar slices. Furthermore, in 3.7% of the cases, a diagnosis of a PNS had been reached in the presence of an atypical IIF pattern without positive line blot typing, warranting caution in regard to the depiction of such IIF screening results as purely clinically irrelevant.

## Additional file

**Additional file 1: Figure S1.** Patient inclusion/exclusion process and number of sample types analyzed.

**Additional file 2: Table S1.** Diagnosis, type of antibody, blot/ titer results and reported patterns of included patients with antineuronal antibodies.
