# Supplementary material for: The clinical significance of atypical indirect immunofluorescence patterns on primate cerebellum in paraneoplastic antibody screening
Source: Auto Immun Highlights. 2019 Jul 25;10(1):6. doi: 10.1186/s13317-019-0116-6 (PMC7065332; doi:10.1186/s13317-019-0116-6)

**Additional file 1: Fig. S1.** Patient inclusion/exclusion process and number of sample types analyzed


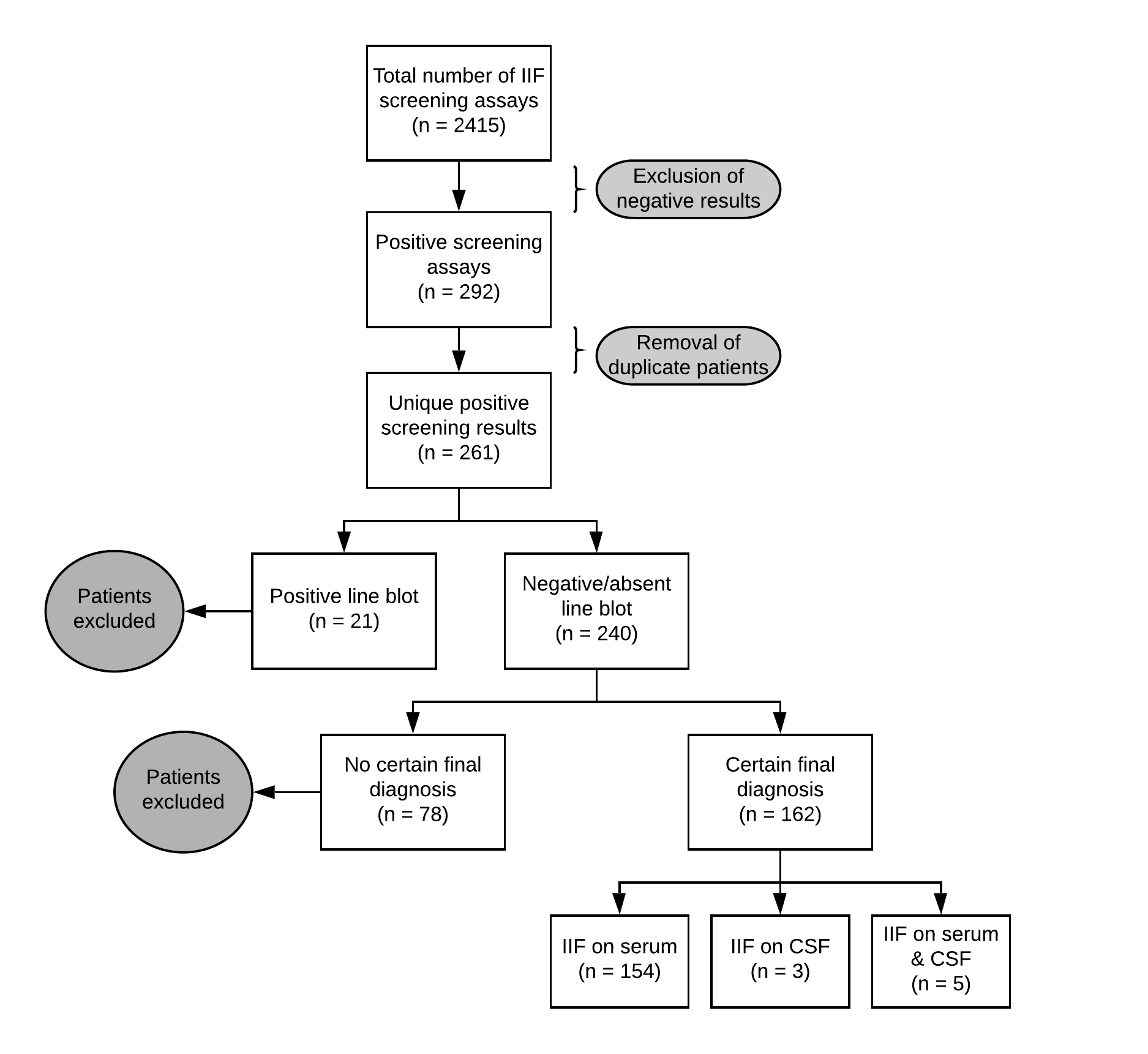

Supplement: Supplementary file 1 — Additional file 1: Figure S1. Patient inclusion/exclusion process and number of sample types analyzed. [file 13317_2019_116_MOESM1_ESM.docx]
