# Supplementary material for: The clinical significance of atypical indirect immunofluorescence patterns on primate cerebellum in paraneoplastic antibody screening
Source: Auto Immun Highlights. 2019 Jul 25;10(1):6. doi: 10.1186/s13317-019-0116-6 (PMC7065332; doi:10.1186/s13317-019-0116-6)
Supplement: Supplementary file 2 — Additional file 2: Table S1. Diagnosis, type of antibody, blot/ titer results and reported patterns of included patients with antineuronal antibodies. [file 13317_2019_116_MOESM2_ESM.docx]

**Article:** The clinical significance of atypical immunofluorescence patterns on primate cerebellum in paraneoplastic antibody screening.

**Journal:** Autoimmunity Highlights

**Authors:** Joris Godelaine^1,2^, Xavier Bossuyt^2,3^, Koen Poesen^1,2^

^1^ Department of Neurosciences, Laboratory for Molecular Neurobiomarker Research, KU Leuven (University of Leuven), Herestraat 49, Leuven, Belgium

^2^ Laboratory Medicine, University Hospitals Leuven, UZ Herestraat 49, Leuven, Belgium

^3^ Department of Microbiology and Immunology, Experimental Laboratory Immunology, KU Leuven (University of Leuven), Leuven, Herestraat 49, Leuven, Belgium.

**Corresponding author:**

Prof. Koen Poesen, koen.poesen@uzleuven.be

**Additional file 2: Table S1.** Diagnosis, type of antibody, blot/ titer results and reported patterns of included patients with antineuronal antibodies

| Antineuronal Ab | Individual diagnosis | Blot/titer result | Pattern reported positive |
| --- | --- | --- | --- |
| **Aquaporine-4 Ab** | Myelitis transversa + NMO | / ^a^ | No pattern reported |
| **Anti-GAD I & II Ab** ^b^ | Neurosarcoidosis | / | Neurofilaments positive |
|  | Temporal lobe epilepsy | / | Multiple patterns reported (molecular & granular layer, synapses of neurons) |
|  | Viral encephalitis | / | Granular layer |
|  | Cerebellar ataxia | + ^c^ | No pattern reported |
|  | Generalized EP | I: ++ , II: +++ | Grey matter |
|  | PERM | +++ | No pattern reported |
|  | SPS | I: + , II: ++ | Neuropil |
|  | SPS | + | No pattern reported |
|  | Temporal lobe epilepsy | ++ | No pattern reported |
|  | Temporal lobe epilepsy | +++ | No pattern reported |
| **Anti-GAD II Ab** | Temporal lobe epilepsy | + | Granular layer |
| **Anti-GAD + Anti-GABA_B_ R Ab** | Temporal lobe EP | GAD I & II: +++, GABA: / | No pattern reported |
|  | *Thymoma: Anti-GAD LE* | GAD II: +++, GABA: / | Grey matter |
| **Anti-VGKC Ab** ^d^ | ALS | 354 pmol/L ^e^ | Granular layer |
|  | Anti-VGKC LE | 855 pmol/L | Molecular layer |
|  | Anti-VGKC LE | 4803 pmol/L | Molecular layer |
|  | Anti-VGKC LE | 189 pmol/L | Granular layer |
|  | Anti-VGKC LE | 188 pmol/L | No pattern reported |
|  | CIDP | 136 pmol/L | Multiple patterns (neuropil, Purkinje cell layer) |
|  | *Lung adenoma: anti-VGKC LE* | / | Molecular layer |
|  | Psychosis | 357 pmol/L | Purkinje cell layer |

*^a^ / = reported positive but blot result (anti-GAD Ab) or titer (AQP-4, Anti-GABA_B_ R Ab, Anti-VGKC Ab) not specified; ^b^ GAD I = GAD 67 kDa, GAD II = 65 kDa; ^c^ If no distinction between GAD I & II is made, the value in the table represents both isoforms; ^d^ Anti-VGKC Ab are nowadays divided/ replaced by anti-LGI1 & anti-CASPR2 Ab. At the time of this study, however, anti-VGKC Ab were still used; ^e^ Reference value anti-VGKC Ab: 0-69 pmol/ L. Individual diagnoses in italics are classified as a paraneoplastic neurological syndrome.*
